# Increased Risks of Suicide Attempt and Suicidal Drug Overdose Following Admission for Head Injury in Patients with Depression

**DOI:** 10.3390/ijerph16193524

**Published:** 2019-09-20

**Authors:** Dorji Harnod, Tomor Harnod, Cheng-Li Lin, Wei-Chih Shen, Chia-Hung Kao

**Affiliations:** 1 Department of Emergency and Critical Care Medicine, Fu Jen Catholic University Hospital, Fu Jen Catholic University, New Taipei City 243, Taiwan; A00635@mail.fjuh.fju.edu.tw; 2 School of Medicine, College of Medicine, Fu Jen Catholic University, New Taipei City 243, Taiwan; 3 Department of Neurosurgery, Hualien Tzu Chi Hospital, Buddhist Tzu Chi Medical Foundation, Hualien 970, Taiwan; 4 College of Medicine, Tzu Chi University, Hualien 970, Taiwan; 5 Management Office for Health Data, China Medical University Hospital, Taichung 404, Taiwan; orangechengli@gmail.com; 6 College of Medicine, China Medical University, Taichung 404, Taiwan; 7 Department of Computer Science and Information Engineering, Asia University, Taichung 413, Taiwan; wcshen@gmail.com; 8 Center of Augmented Intelligence in Healthcare, China Medical University Hospital, Taichung 404, Taiwan; 9 Graduate Institute of Biomedical Sciences and School of Medicine, College of Medicine, China Medical University, Taichung 404, Taiwan; 10 Department of Nuclear Medicine and PET Center, China Medical University Hospital, Taichung 404, Taiwan; 11 Department of Bioinformatics and Medical Engineering, Asia University, Taichung 413, Taiwan

**Keywords:** cohort study, depression, suicide, head injury, National Health Insurance

## Abstract

Aims: To determine the risks of suicide attempt (SA) and suicidal drug overdose (SDO) following the admission for head injury of patients with depression. Design: We analyzed the NHIRD data of patients aged ≥20 years who had received depression diagnoses between 2000 and 2010. They were divided into cohorts of those with admission for head injury (DHI) and those without it (DWI) during the follow-up period and compared against a sex-, age-, comorbidity-, and index-date-matched cohort from the general population. Setting: The Taiwan National Health Insurance Research Database (NHIRD). Participants/Cases: We analyzed the NHIRD data of patients (≥20 years) who had received depression diagnoses between 2000 and 2010. Intervention(s): Regular interventions. Measurements: We calculated the adjusted hazard ratios (aHRs) and 95% confidence intervals (CIs) of SA and SDO in these cohorts after adjustment for age, sex, and comorbidities. Findings: Up to the end of 2011, our results revealed extremely high incidences of SA and SDO with 63.3 and 88.6 per 10,000 person-years, respectively, in the DHI cohort. The DHI cohort had a 37.4-times higher risk for SA and a 17.1-times higher risk for SDO compared with the comparison group and had aHRs of 14.4 and 16.3, respectively, for poisoning by medicinal substances and poisoning by tranquilizers compared with patients in the DWI cohort. Patients with DHI aged <50 years, of female sex, with high incomes, living in more urbanized areas, and without other comorbidities had extraordinarily higher risks for SA. Conclusions: The risks of SA and SDO were proportionally increased by head injury in patients with depression in Taiwan. Our findings provide crucial information to implement efficient suicide prevention strategies in the future.

## 1. Introduction

Depression is a common brain disorder worldwide and is characterized by key symptoms of low and depressed mood, loss of interest and pleasure, fatigue, and reduced energy for at least 2 weeks [1]. Depression can be a solitary disorder with an idiopathic cause, or it can be coexisting with another disease, which increases the complexity and care burden of the depression itself. The prevalence rate of coexisting depression in patients with various neurological disorders, such as stroke, multiple sclerosis, Parkinson’s disease, or epilepsy, is 20–50% [2]. In addition, patients with depression usually experience low self-confidence, guilt feeling with self-blame, and suicidal ideation. Depression is thus known as the main psychiatric disorder associated with suicidal behavior or suicide attempt (SA) [3,4]. However, depression is usually underdiagnosed and undertreated even in developed Western countries [5,6]. Moreover, one of five adults has considered suicide in United Kingdom and one in 15 has attempted suicide [7]. Thus, the majority of patients at a high risk of suicide will not die by suicide. Instead of the low predictive value and efficiency by finding the ones with suicide ideation, identifying the ones who at a high risk for attempting suicide and who might die by suicide should be the priority for patient safety and suicide prevention among individuals with depression [7].

Even in the 21st century, head injury remains one of the leading causes of death worldwide, irrespective of Western or Asian countries [8,9]. In a study including 48,792 Taiwanese patients admitted with moderate or severe head injury, 4935 patients died, and the standardized in-hospital mortality rate for head injury was 10.7 deaths per 100,000 person-years in Taiwan [10]. However, head injury may result in various degrees of paralysis, consciousness impairment, and cognitive problems other than death. Patients surviving an initial admission for head injury often experience long-term or even life-long emotional and mental consequences [11,12,13], which can probably increase the severity of depression and the risk of SA [14,15]. However, it remains unclear to what extent the risk of SA is increased in patients with depression following a head injury. We believe that a more thorough understanding of the risk and risk factors for suicidal behavior in patients with depression plays a key role in building an effective strategy for suicide prevention.

Taiwan is located in East Asia and has similar mores to those of China and most of Southeast Asian societies [16]. The Taiwanese government has run an established nationwide health care system for 2 decades [17]. This study used the Taiwan National Health Insurance Research Database (NHIRD) to determine whether the risk of SA was increased in patients with depression admitted for head injury. Because of the differences in cultural, ethnic, and socioeconomic backgrounds between Taiwanese and Western societies [16,18] our data regarding the risk of SA in Taiwanese patients might not be applicable to Western countries. However, these data could be beneficial both for the Taiwanese clinicians and government and for other Asian countries.

## 2. Methods

### 2.1. Data Source

In 1995, Taiwan government implemented a nationwide database (NHIRD), which includes the medical records of all beneficiaries of the single-payer health care system in Taiwan. Medical records include the history of outpatient visits, hospitalizations, prescriptions of medication, and other medical services. As of today, more than 99% of residents are enrolled in the database [17]. To ensure the high validity of this study, we used hospitalization files from the NHIRD. All study participants were patients with a hospitalization record. The identification number of each study participant was recoded before the database was released by the government. Diagnoses were made according to the International Classification of Diseases, Ninth Revision, Clinical Modification (ICD-9-CM) coding system. The Research Ethics Committee of the China Medical University and Hospital in Taiwan approved the study (CMUH104-REC2-115-CR4).

### 2.2. Study Population

To clarify the association between patients with depression admitted for head injury and the risk of SA and suicidal drug overdose (SDO), we included patients aged 20 or more and defined four cohorts as follows: patients with depression (ICD-9 CM: 296.2, 296.3, 296.82, 300.4, and 311) constituted the total depression cohort; patients with depression and admitted for head injury (ICD-9 CM: 850–854 and 959.01) represented the DHI group; and patients with depression but without head injury were part of the DWI group. Patients without depression or head injury between 2000 and 2010 were selected as the comparison cohort. The date a patient received head injury or depression diagnosis was defined as the index date in the DHI and DWI cohorts. The comparison cohort was frequency-matched by sex, age, and index year.

### 2.3. Outcome Measurement

The primary outcome in this study was the occurrence of SA (ICD-9-CM: E950–E959). To further understand the risk of SA and the safety of long-term prescription in patients with depression, both the SDO and SA in these cohorts were analyzed. SDO was defined as hospitalization with the ICD-9-CM codes 960–979 but without E codes. We excluded patients younger than 20 years and those who experienced SA or SDO before the index date. The covariates included a history of schizophrenia (ICD-9 CM: 295), alcohol-related illness (ICD-9 CM: 291, 303, 305.00, 305.01, 305.02, 305.03, 571.0, 571.1, 571.3, 790.3, and V11.3), anxiety (ICD-9 CM: 300.00), mental disorders (ICD-9 CM: 290–319), insomnia (ICD-9 CM: 307.4 and 780.5), diabetes mellitus (ICD-9 CM: 250), hypertension (ICD-9 CM: 401–405), hyperlipidemia (ICD-9 CM: 272), chronic obstructive pulmonary disease (COPD) (ICD-9 CM: 491, 492 and 496), coronary artery disease (CAD) (ICD-9 CM: 410–414), stroke (ICD-9 CM: 430-–38), and cirrhosis (ICD-9 CM: 571, 572), which were all defined by at least one hospitalization record before the index date. The follow-up period for the study participants began at the index date and lasted until the date of SA or SDO, death, withdrawal from the NHIRD, or December 31, 2011, whichever came first.

### 2.4. Statistical Analysis

In this study, we expressed the demographic factors and mean age by number (%) and mean (standard deviation (SD)) for the categorical and continuous variables, respectively. The differences in variables among the four cohorts were tested using the chi-square test for categorical variables and analysis of variance for continuous variables. The incidence rates of SA and SDO were calculated by dividing the SA or SDO events by the person-years (every 10,000 person-years). The Kaplan–Meier method was applied to demonstrate the cumulative incidence curve of development of SA and SDO in the DHI, DWI, and comparison cohorts, and the differences between the cumulative incidence curves were established using the log-rank test. The risks of SA and SDO in the total depression, DHI, DWI, and comparison cohorts were evaluated using crude and adjusted Cox proportional hazard models and presented as hazard ratios (HRs), adjusted HRs (aHRs), and 95% confidence intervals (CIs). The statistical analyses were conducted with type I error α = 0.05 using a statistical software package, SAS, version 9.4 (SAS Institute, Inc, Cary, NC, USA).

## 3. Results

In this study, we identified the following four cohorts: total depression (*n* = 669,244), DWI (*n* = 647,375), DHI (*n* = 21,869), and comparison (*n* = 1,338,487) cohorts. After frequency matching by sex, age, and index year, no significant differences were observed among the four groups (*p* = 0.99). Significant differences were noted among the four cohorts in the monthly income, urbanization level, occupation, and comorbidities (*p* < 0.001). Of the participants constituting the overall study cohorts, 60.1% were men, and 39.9% were women. The mean (SD) age was 46.8 (19.3) years in the total depression (DHI + DWI) cohort and 46.6 (19.3) years in the comparison cohorts (Table 1).

**Figure 1:**
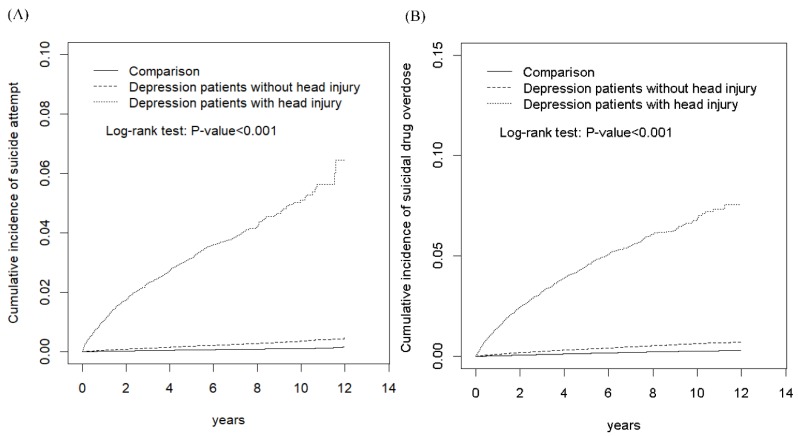
Comparison of the cumulative incidence of (**A**) suicide attempt and (**B**) suicidal drug overdose among patients with depression with head injury, patients with depression without head injury, and comparison group from the general population.

Figure 1 presents the cumulative incidence curves of SA and SDO in the DHI, DWI, and comparison cohorts. The results revealed significant differences in the cumulative incidences of SA between the DHI, DWI, and comparison cohorts, with a *p* value for the log-rank test of <0.001 (Figure 1A). Moreover, the results indicated significant differences in the cumulative incidences of SDO between the DWI, DHI, and comparison cohorts, with a *p* value for the log-rank test of <0.001 (Figure 1B).

Table 2 exhibits the event numbers, incidences, HRs, and 95% CIs for distinct risk factors associated with SA. The incidences of SA in the comparison, total depression, DWI, and DHI cohorts were 1.16, 5.50, 3.76, and 63.3 per 10,000 person-years, respectively. Compared with each comparison group, patients in the DHI cohort (aHR = 37.4, 95% CI = 32.9–42.4) and aged less than 50 (aHR = 1.45, 95% CI = 1.30–1.62) had specifically significantly higher risks of SA than others. Patients with monthly income of less than New Taiwan Dollar (NTD) 15,000 (aHR = 1.66, 95% CI = 1.44–1.90), with a monthly income of NTD 15,000–19,999 (aHR = 1.56, 95% CI = 1.38–1.76), living in urbanization level two, three, and four areas (aHR = 1.33, 1.51, 1.64; 95% CI = 1.18–1.50, 1.32–1.72, 1.46–1.85), with a laborer occupation (aHR = 1.24, 95% CI = 1.13–1.36), with other occupations (aHR = 1.29, 95% CI = 1.13–1.36), and with comorbidities of schizophrenia (aHR = 3.28, 95% CI = 2.61–4.11), alcohol-related illness (aHR = 3.80, 95% CI = 3.16–4.56), anxiety (aHR = 2.32, 95% CI = 1.82–2.96), mental disorder (aHR = 0.71, 95% CI = 0.58–0.87), insomnia (aHR = 2.37, 95% CI = 1.96–2.85), diabetes (aHR = 1.31, 95% CI = 1.13–1.51), hyperlipidemia (aHR = 1.36, 95% CI = 1.15–1.60), COPD (aHR = 1.22, 95% CI = 1.03–1.45), and cirrhosis (aHR = 1.26, 95% CI = 1.07–1.47) had significantly higher risks of SA after adjustment for demographic factors and comorbidities (Table 2).

Table 3 presents the analysis results of SA risk stratified by sex, age, monthly income, urbanization level, occupation, comorbidities, and compared with the comparison cohorts. Patients aged less than 50, aged 50–64, aged ≥65 years, of female sex, and male sex all had significantly higher risks of SA. When stratified with monthly income, urbanization level, and occupation, patients with monthly income of less than NTD 15,000, monthly income of NTD 15,000–19,999, monthly income of ≥NTD 20,000, living in urbanization level one to four areas, working as office workers, laborers, and other occupations all had significantly higher risks of SA in the total depression, DWI, and DHI cohorts. Both the patients with any one of the comorbidities and without had significantly higher risks of SA in the total depression, DWI, and DHI cohorts. When specifically considering the enhancing effects of admission for head injury and depression on the DHI cohort, patients aged <50 years, of female sex, and without other comorbidity had extremely higher risks of SA than others (Table 3).

Because we determined that admission for head injury had a significantly enhancing effect on SA in patients with depression, we further compared the DHI cohort to the DWI cohort, and the results are presented in Table 4. We noted that patients in the DHI cohort (aHR = 14.8, 95% CI = 13.2–16.7), aged <50 years (aHR = 18.8, 95% CI = 16.4–21.5), of female sex (aHR = 21.7, 95% CI = 18.4–25.7), and without any comorbidity (aHR = 22.7, 95% CI = 19.6–26.4) had specifically significantly higher risks of SA. When considered the monthly income, urbanization, and occupation of patients, the ones with monthly income of less than NTD 15000, monthly income of NTD 15000-19999, and monthly income of ≥NTD 20000; the ones living in urbanization level one areas, level two areas, level three areas, and level four areas; who are office workers, laborers, and in other occupations all had significantly higher risks of SA (Table 4).

The total depression, DWI, and DHI cohorts appeared to exhibit positive associations with SDO after adjustment for age, monthly income, urbanization level, and comorbidities. The incidences of SDO in the comparison, total depression, DWI, and DHI cohorts were 2.64, 9.34, 6.97, and 88.6 per 10,000 person-years, respectively. Compared with the comparison cohort, patients had significantly higher risks for SDO in the total depression (aHR = 2.71, 95% CI = 2.55–2.88), DWI (aHR = 2.20, 95% CI = 2.06–2.34), and DHI (aHR = 17.1, 95% CI = 15.5–18.8) cohorts. When compared with the DWI cohort, the DHI cohort had a significantly higher risk of SDO (aHR = 9.04, 95% CI = 8.22–9.94) (Table 5). In addition, we noted that the DHI cohort exhibited higher risks of SDO when combined with poisoning by medicinal substances (aHR = 14.4, 95% CI = 11.4–18.3), tranquilizers (aHR = 16.3, 95% CI = 13.3–19.9), and others (aHR = 6.68, 95% CI = 5.91–7.55) than did the DWI cohort.

## 4. Discussion

In this study including participants aged ≥20 years and with a 12-year follow-up period, we observed that 3.27% (21,869 out of 669,244) of patients with depression were admitted for head injury. After admission for head injury, patients had a high (37.4 times) risk of SA compared with the patients without head injury who had a moderately increased (2.76 times) risk of SA. Moreover, patients with head injury had a following aHR of SDO as high as 17.1 compared to those in the comparison group; the DHI cohort had higher aHRs of 9.04, 14.4, and 16.3 for all SDO, poisoning by medicinal substances, and poisoning by tranquilizers, respectively, compared to the DWI cohort. Our observation in this study revealed evident proportional increases in the risks of SA and SDO in the patients with depression and head injury. This implied that medical substances and tranquilizers taken from the medical care system could be dangerously abused for SA (by taking a high dose of medications at one time) by patients with depression and head injury. A long-term prescription containing large amounts of medications should be carefully attributed for patients with coexisting depression and head injury in the future.

Apart from the known standardized hospitalization death rate of 10.7 deaths per 100,000 person-years in Taiwanese patients with traumatic head injury only [10], this study revealed other risks in patients with depression and head injury, namely SA and SDO risks. The incidences of SA and SDO were 63.3 and 88.6 per 10,000 person-years, respectively, in patients with depression surviving from a head injury. Furthermore, our data in this study implied that their suicide risk could cumulatively increase in a decade or more. Therefore, we suggest that admission for head injury should be considered a serious disorder when interacting with patients with depression and highly dangerous for suicidal behaviors in these patients. In the literature, most previous studies have been designed to evaluate the suicidal risk mediated by the development of depression in patients surviving head injury [19,20,21]. Increasing awareness of depression in the population with head injury would help identify at-risk individuals, particularly because of the current general belief that depression is a common sequela after traumatic brain injury. To the best of our knowledge, this study provided the first nationwide population-based evidence of the risks of suicide attempt and drug overdose following a head injury in patients with depression. We documented the overwhelming enhancement of SA and SDO deriving from the admission for head injury in patients with depression for the first time.

According to Taiwan NHI guidelines, if a patient experiences a head injury with clear consciousness and does not have any intracranial hemorrhage or brain contusion based on brain images, the patient’s head injury is classified as mild and as one to be treated in an outpatient service. Therefore, patients admitted for head injuries in this study were classified as moderate or severe with promising brain injuries or insults. The effects of depression and admission for head injury on suicidal behaviors and how they interact to increase the risks of SA and SDO require clarification. From the aspect of neuro-psychological factors of an individual with head injury, effects of various factors such as chronic cognitive and physical fatigue and associated psychiatric, sleep, or psychosocial sequelae are usually developed in patients [21,22,23]. Post-traumatic amnesia, poor attention, impaired processing speed, and anxiety were noted and led to a pre- and post-injury gap in patients with pre-existing depression. Patients have difficulties re-engaging in the desired living activities and are more likely to experience anxiety, which in turn may contribute to suicidal behaviors. In addition, biological evidence has demonstrated that head injury with brain insult could increase the neuro-inflammation pathologies over the blood–brain barrier, glutamate regulation, microglia activation, and even autoimmune response [24,25]. The elevation of the levels of pro-inflammatory cytokines has been documented in patients with major depressive disorder and bipolar disorder with the induction of depressive symptomatology. All of those conditions facilitate the further development of depression and other mental disorders, which are associated with suicidality.

SDO is a nonviolent suicide method with a generally lower mortality rate than suicide by violent means. Because of the restricted access to firearms in most Asian countries, Asian patients may find it easier to attempt suicide through the nonviolent method of self-poisoning [26,27]. This study reminds us that self-poisoning with medical substances or tranquilizers is the most accessible method for attempting suicide in patients with depression and head injury. Moreover, when implementing suicide prevention systems in the society, age <50 years, female sex, and the absence of other comorbidity could be thought of as high-risk factors associating with SA. When compared with a similar study in Finland [28], we considered that monthly income and urbanization level were controversial effects for the risk of SA in Taiwan. Our findings are valuable for clinicians and governments in developing Asian countries with a heritage similar to that of Taiwan.

However, this study had several limitations. First, this was a retrospective study that included all the NHI claims data on hospitalizations related to head injury and depression during the study period. The accuracy of NHI claims data is assured by the severe penalty incurred for fraud and false claiming. We identified suicide behaviors, head injury, and depression on the basis of the ICD-9-CM coding system. Patients with minor SA or SDO, head injury, or depression may not report to the medical care system, although the patients with mild head trauma have also been documented to be associated with depression and suicidality [29]. This might result in underestimation of the risks of SA and SDO. Second, because patients’ identities were anonymized in the NHIRD, we could not directly understand the details of patients’ disorders, for example, the Glasgow Coma Scale or the severity score during the admission for head injury, the site and number of brain injury, or whether the initial head injury was due to self-harm of a SA in this study. Patients with frontal lobe injury or multiple brain injuries would have more psychological deficits than those with a single parietal lobe injury. The other issue is that the details of personal psychosocial factors could not be accessed and analyzed, such as individual alienation from family and society, suicide ideation, severity of personal stress, severity of depression, and other frustrations with performance after the head injury. In addition, painkillers or other medications used for head injury and depression might lead to abuse and be involved in patients’ suicidality. Those are potential confounders associated with the risks of suicide behaviors. Finally, although our study design already controlled for numerous confounding factors, to our knowledge, some unmeasured or unknown confounders may have remained. However, this large-scale, fairly unselective study has sufficiently evidenced to be hedged against the limitations and other confounding variables above. Patients with coexistence of depression and admission for head injury should be considered at a high risk for suicide attempt and drug overdose. They might need more concern and treatment by physicians for patient safety and suicide prevention.

## 5. Conclusions

The risks of SA and SDO were proportionally increased by head injury in patients with depression in Taiwan. Our findings provide crucial information for clinicians and the government in Taiwan for implementing suicide prevention strategies. Moreover, whether it is adequate to offer long-term medical substances or tranquilizers to patients with depression admitted for head injury should be seriously questioned in the future. It is necessary to conduct more large-scale studies to clarify the effect of coexisting depression and head injury on suicidality worldwide.

## Figures and Tables

**Table 1:** Distribution of age, sex, and comorbidities between the depression and comparison cohorts.

	Total Depression *n* = 669,244		Depression without Head Injury *n* = 647,375		Depression with Head Injury *n* = 21,869		Comparison Cohort *n* = 1,338,487		
Variable	*n*	%	*n*	%	*n*	%	*n*	%	*p*-value ^a^
Sex									0.99
Women	266,818	39.9	256,988	39.7	9830	45.0	533,635	39.9	
Men	402,426	60.1	390,387	60.3	12,039	55.1	804,852	60.1	
Age stratified									0.99
≤49	392,928	58.7	382,001	59.0	10,927	50.0	785,856	58.7	
50–64	135,145	20.2	130,542	20.2	4603	21.1	270,290	20.2	
≥65	141,171	21.1	134,832	20.8	6339	29.0	282,341	21.1	
Age, mean ± SD ^§^	46.8 ± 19.3		46.6 ± 19.3		51.9 ± 19.0		46.6 ± 19.3		<0.001
Monthly income ^†^									<0.001
<15,000	186,695	27.9	179,296	27.7	7399	33.8	368,820	27.6	
15,000–19,999	350,534	52.4	339,618	52.5	10,916	49.9	595,878	44.5	
≥20000	132,015	19.7	128,461	19.8	3554	16.3	373,789	27.9	
Urbanization level ^‡^									<0.001
1 (highest)	130,588	19.5	126,385	19.5	4203	19.2	397,048	29.7	
2	200,516	30.0	193,631	29.9	6885	31.5	407,762	30.5	
3	122,856	18.4	119,185	18.4	3671	16.8	233,662	17.5	
4 (lowest)	215,284	32.2	208,174	32.2	7110	32.5	300,015	22.4	
Occupation category ^b^									<0.001
Office worker	293,059	43.8	284,726	44.0	8333	38.1	722,439	54.0	
Laborer	296,329	44.3	286,725	44.3	9604	43.9	472,903	35.3	
Other	79,856	11.9	75,924	11.7	3932	18.0	143,145	10.7	
Comorbidity									
Schizophrenia	4752	0.71	3896	0.60	856	3.91	5091	0.38	<0.001
Alcohol-related illness	20,923	3.13	16,698	2.58	4225	19.3	4774	0.36	<0.001
Anxiety	5922	0.88	4030	0.62	1892	8.65	3080	0.23	<0.001
Mental disorders	32,178	4.81	32,178	4.97	-	-	13,176	0.98	<0.001
Insomnia	12,875	1.92	9428	1.46	3447	15.8	7584	0.57	<0.001
Diabetes mellitus	62,981	9.41	58,629	9.06	4352	19.9	54,234	4.05	<0.001
Hypertension	102,212	15.3	94,411	14.6	7801	35.7	100,118	7.48	<0.001
Hyperlipidemia	29,557	4.42	26,260	4.06	3297	15.1	25,722	1.92	<0.001
Chronic obstructive pulmonary disease	36,755	5.49	33,269	5.14	3486	15.9	33,050	2.47	<0.001
Coronary artery disease	42,354	6.33	38,133	5.89	4221	19.3	46,876	3.50	<0.001
Stroke	51,657	7.72	46,567	7.19	5090	23.3	41,756	3.12	<0.001
Cirrhosis	45,135	6.74	40,106	6.20	5029	23.0	27,383	2.05	<0.001

Chi-square test; ^§^ *t*-test; ^a^ total depression vs. comparison cohort; ^†^ New Taiwan Dollar (NTD), 1 NTD is equal to 0.03 USD; ^‡^ the urbanization levels were divided according to the population density of the residential area into four levels: level one was the most urbanized and level four was the least urbanized; ^b^ the other occupation category included primarily retired, unemployed, and low-income populations.

**Table 2:** Incidences and risk factors associated with various factors for suicide attempt.

Variable	Event	PY	Rate ^#^	Crude HR (95% CI)	Adjusted HR (95% CI) ^$^
Depression					
None	796	6,850,494	1.16	1.00	1.00
Total depression	1832	3,328,169	5.50	4.73 (4.36-5.14) ***	3.64 (3.34-3.97) ***
Depression without head injury	1216	3,230,837	3.76	3.24 (2.96-3.54) ***	2.76 (2.52-3.02) ***
Depression with head injury	616	97,331	63.3	53.9 (48.5-59.9) ***	37.4 (32.9-42.4) ***
Age group, years					
≤49	1746	6,385,103	2.73	1.36 (1.22-1.51) ***	1.45 (1.30-1.62) ***
50–64	413	2,029,431	2.04	1.00	1.00
≥65	469	1,764,128	2.66	1.28 (1.12-1.46) ***	1.10 (0.96-1.26)
Sex					
Women	1007	4,063,886	2.48	1.00	1.00
Men	1621	6,114,777	2.65	1.07 (0.99-1.16)	
Monthly income ^†^					
<15,000	813	2,661,854	3.05	2.28 (2.02-2.59) ***	1.66 (1.44-1.90) ***
15,000-19,999	1459	4,824,143	3.02	2.28 (2.03-2.56) ***	1.56 (1.38-1.76) ***
≥20,000	356	2,692,666	1.32	1.00	1.00
Urbanization level ^‡^					
1 (highest)	402	2,700,412	1.49	1.00	1.00
2	755	3,105,454	2.43	1.63 (1.45-1.84) ***	1.33 (1.18-1.50) ***
3	511	1,800,161	2.84	1.91 (1.67-2.17) ***	1.51 (1.3-1.72) ***
4 (lowest)	960	2,572,636	3.73	2.50 (2.23-2.81) ***	1.64 (1.46-1.85) ***
Occupation category ^b^					
Office worker	988	5,198,281	1.90	1.00	1.00
Laborer	1242	3,902,147	3.18	1.67 (1.54-1.82) ***	1.24 (1.13-1.36) ***
Other	398	1,078,233	3.69	1.93 (1.72-2.17) ***	1.29 (1.13-1.47) ***
Comorbidity					
Schizophrenia					
No	2546	10,131,823	2.51	1.00	1.00
Yes	82	46,839	17.5	6.91 (5.55-8.61) ***	3.28 (2.61-4.11) ***
Alcohol-related illness					
No	2361	10,070,372	2.34	1.00	1.00
Yes	267	108,291	24.7	10.3 (9.08-11.7) ***	3.80 (3.16-4.56) ***
Anxiety					
No	2547	10,141,437	2.51	1.00	1.00
Yes	81	37,226	21.8	8.44 (6.76-10.5) ***	2.32 (1.82-2.96) ***
Mental disorders					
No	2508	10,002,488	2.51	1.00	1.00
Yes	120	176,174	6.81	2.63 (2.19-3.16) ***	0.71 (0.58-0.87) **
Insomnia					
No	2480	10,099,868	2.46	1.00	1.00
Yes	148	78,794	18.8	7.39 (6.26-8.73) ***	2.37 (1.96-2.85) ***
Diabetes mellitus					
No	2366	9,737,101	2.43	1.00	1.00
Yes	262	441,561	5.93	2.35 (2.07-2.67) ***	1.31 (1.13-1.51) ***
Hypertension					
No	2255	9,417,225	2.39	1.00	1.00
Yes	373	761,437	4.90	1.97 (1.76-2.20) ***	1.13 (0.98-1.30)
Hyperlipidemia					
No	2436	9,954,581	2.45	1.00	1.00
Yes	192	224,081	8.57	3.40 (2.94-3.94) ***	1.36 (1.15-1.60) ***
Chronic obstructive pulmonary disease					
No	2457	9,921,558	2.48	1.00	1.00
Yes	171	257,104	6.65	2.58 (2.21-3.02) ***	1.22 (1.03-1.45) *
Coronary artery disease					
No	2437	9,839,848	2.48	1.00	1.00
Yes	191	338,814	5.64	2.20 (1.90-2.55) ***	1.16 (0.97-1.38)
Stroke					
No	2457	9,840,840	2.50	1.00	1.00
Yes	171	337,822	5.06	1.94 (1.66-2.27) ***	0.94 (0.79-1.13)
Cirrhosis					
No	2301	9,878,155	2.33	1.00	1.00
Yes	327	300,507	10.9	4.57 (4.07-5.13) ***	1.26 (1.07-1.47) **

CI, confidence interval; HR, hazard ratio; PY, person-years; ^#^ incidence rate per 10,000 person-years; ^$^ multivariable analysis included age, monthly income, urbanization level, and comorbidities of schizophrenia, alcohol-related illness, anxiety, mental disorders, insomnia, diabetes mellitus, hypertension, hyperlipidemia, chronic obstructive pulmonary disease, coronary artery disease, stroke, and cirrhosis; ^†^ New Taiwan Dollar (NTD), 1 NTD is equal to 0.03 USD; ^‡^ the urbanization levels were divided according to the population density of the residential area into four levels: level one was the most urbanized, and level four was the least urbanized; ^b^ the other occupation category included primarily retired, unemployed, and low-income populations; * *p* < 0.05, ** *p* < 0.01, *** *p* < 0.001.

**Table 3:** Comparison of hazard ratios for suicide attempt stratified by age, sex, and comorbidity between the depression and comparison cohorts.

	Comparisonn *n* = 1,338,487		Total Depression *n* = 669,244			Depression without Head Injury *n* = 647,375			Depression with Head Injury *n* = 21,869		
Variable	Event (*n*)	Rate ^#^	Event (*n*)	Rate	Adjusted HR (95% CI) ^$^	Event (*n*)	Rate	Adjusted HR (95% CI)	Event (*n*)	Rate	Adjusted HR (95% CI)
Age, years											
≤49	434	1.02	1312	6.14	4.62 (4.13-5.17) ***	832	4.00	3.36 (2.98-3.78) ***	480	88.1	60.7 (52.1-70.6) ***
50–64	123	0.89	290	4.43	3.45 (2.76-4.30) ***	206	3.25	2.82 (2.24-3.55) ***	84	40.5	24.8 (17.7-34.7) ***
≥65	239	1.95	230	4.28	1.81 (1.50-2.19) ***	178	3.45	1.54 (1.26-1.88) ***	52	23.5	8.09 (5.72-11.4) ***
*p* for interaction					<0.001			<0.001			
Sex											
Female	267	0.98	740	5.51	4.57 (3.95-5.27) ***	420	3.24	2.98 (2.55-3.49) ***	320	69.4	60.1 (49.9-72.3) ***
Male	529	1.28	1092	5.50	3.15 (2.82-3.51) ***	796	4.12	2.62 (2.34-2.93) ***	296	57.8	25.0 (20.9-30.0) ***
*p* for interaction					0.003			<0.001			
Monthly income ^†^											
<15,000	267	1.49	546	6.26	3.41 (2.92-3.97) ***	372	4.42	2.65 (2.2-3.12) ***	174	55.6	26.6 (21.1-33.5) ***
15,000–19,999	414	1.36	1045	5.88	3.43 (3.05-3.86) ***	702	4.06	2.62 (2.32-2.97) ***	343	68.9	36.3 (30.6-43.0) ***
≥20,000	115	0.57	241	3.56	4.55 (3.61-5.74) ***	142	2.15	3.14 (2.44-4.04) ***	99	61.1	78.2 (56.5-108.3) ***
*p* for interaction					0.004			<0.001			
Urbanization level ^‡^											
1 (highest)	150	0.73	252	3.88	4.03 (3.27-4.98) ***	146	2.31	2.70 (2.13-3.41) ***	106	56.9	52.0 (2.13-3.41) ***
2	220	1.05	535	5.32	4.01 (3.41-4.72) ***	329	3.37	2.85 (2.40-3.39) ***	206	66.5	46.7 (37.3-58.5) ***
3	166	1.40	345	5.59	3.29 (2.72-3.98) ***	246	4.10	2.65 (2.16-3.23) ***	99	58.8	28.2 (21.0-38.1) ***
4 (lowest)	260	1.71	700	6.63	3.23 (2.79-3.74) ***	495	4.83	2.60 (2.24-3.03) **	205	66.4	28.8 (23.1-35.8) ***
*p* for interaction					0.001			<0.001			
Occupation category ^b^											
Office worker	314	0.84	674	4.56	4.13 (3.59-4.74) ***	442	3.07	3.11 (2.68-3.61) ***	232	61.2	49.4 (40.2-60.7) ***
Laborer	353	1.45	889	6.03	3.36 (2.96-3.81) ***	602	4.21	2.57 (2.25-2.94) ***	287	66.7	34.3 (28.5-41.2) ***
Other	129	1.84	269	7.14	2.97 (2.39-3.71) ***	172	4.77	2.21 (1.75-2.80) ***	97	59.2	22.6 (16.5-31.1) ***
*p* for interaction					0.002			<0.001			
Comorbidity											
None	581	0.95	997	4.06	3.80 (3.43-4.22) ***	774	3.19	2.99 (2.68-3.34) ***	223	74.3	68.0 (58.2-79.4) ***
With any one	215	2.96	835	9.60	2.57 (2.20-2.99) ***	442	5.51	1.53 (1.30-1.81) ***	393	58.4	14.8 (12.4-17.5) ***
*p* for interaction					0.003			0.05			

CI, confidence interval; HR, hazard ratio; PY, person-years; ^#^ incidence rate per 10,000 person-years; ^$^ multivariable analysis included age, monthly income, urbanization level, and comorbidities of schizophrenia, alcohol-related illness, anxiety, mental disorders, insomnia, diabetes mellitus, hypertension, hyperlipidemia, chronic obstructive pulmonary disease, coronary artery disease, stroke, and cirrhosis; ^†^ New Taiwan Dollar (NTD), 1 NTD is equal to 0.03 USD; ^‡^ the urbanization levels were divided according to the population density of the residential area into four levels: level one was the most urbanized, and level four was the least urbanized; ^b^ the other occupation category included primarily retired, unemployed, and low-income populations; ^§^ individuals with schizophrenia, depression, alcohol-related illness, anxiety, mental disorders, and insomnia were classified into the comorbidity group; ** *p* < 0.01, *** *p* < 0.001.

**Table 4:** Comparison of incidences and hazard ratios for suicide attempt stratified by age, sex, and comorbidities between patients with depression with and without head injury.

	Depression without Head Injury *n* = 647,375	Depression with Head Injury *n* = 21,869
Variable	Adjusted HR ^$^ (95% CI)	Adjusted HR ^$^ (95% CI)
All	1.00	14.8 (13.2-16.7) ***
Age, years		
≤49	1.00	18.8 (16.4-21.5) ***
50–64	1.00	9.62 (7.07-13.1) ***
≥65	1.00	6.00 (4.21-8.57) ***
*p* for interaction		<0.001
Sex		
Female	1.00	21.7 (18.4-25.7) ***
Male	1.00	10.6 (8.92-12.5) ***
*p* for interaction		<0.001
Monthly income ^†^		
<15000	1.00	11.3 (9.08-14.0) ***
15000–19999	1.00	14.9 (12.7-17.4) ***
≥20000	1.00	26.1 (19.2-35.6) ***
*p* for interaction		<0.001
Urbanization level ^‡^		
1 (highest)	1.00	22.0 (16.3-29.7) ***
2	1.00	17.5 (14.2-21.5) ***
3	1.00	12.5 (9.45-16.5) ***
4 (lowest)	1.00	11.8 (9.70-14.5) ***
*p* for interaction		<0.001
Occupation category ^b^		
Office worker	1.00	17.8 (14.7-21.6) ***
Laborer	1.00	14.2 (12.0-16.8) ***
Other	1.00	12.0 (8.27-15.2) ***
*p* for interaction		<0.001
Comorbidity ^§^		
None	1.00	22.7 (19.6- 26.4) ***
With any one	1.00	9.54 (8.32-10.9) ***
*p* for interaction		<0.001

CI, confidence interval; HR, hazard ratio; ^$^ multivariable analysis included age, monthly income, urbanization level, and comorbidities of schizophrenia, alcohol-related illness, anxiety, mental disorders, insomnia, diabetes mellitus, hypertension, hyperlipidemia, chronic obstructive pulmonary disease, coronary artery disease, stroke, and cirrhosis; ^†^ New Taiwan Dollar (NTD), 1 NTD is equal to 0.03 USD; ^‡^ the urbanization levels were divided according to the population density of the residential area into four levels: level one was the most urbanized areas, and level four was the least urbanized areas; ^b^ the other occupation category included primarily retired, unemployed, and low-income populations; ^§^ individuals with schizophrenia, depression, alcohol-related illness, anxiety, mental disorders, and insomnia were classified into the comorbidity group; ** *p* < 0.01, *** *p* < 0.001.

**Table 5:** Incidences and hazard ratios of suicidal drug overdose (per 10,000 person-years) in patients with depression with or without head injury using the Cox method.

	Comparison Cohort	Total Depression	Depression without Head Injury	Depression with Head Injury
	(*n* = 647375)	(*n* = 669,244)	(*n* = 647,375)	(*n* = 21,869)
Person-years				
Event, *n*	1810	3104	2251	853
Rate	2.64	9.34	6.97	88.6
Crude HR (95% CI)	1 (Reference)	3.53 (3.33-3.74) ***	2.64 (2.48-2.80) ***	33.1 (30.5-35.9) ***
Adjusted HR ^$^ (95% CI)	1 (Reference)	2.71 (2.55-2.88) ***	2.20 (2.06-2.34) ***	17.1 (15.5-18.8) ***
Crude HR (95% CI)			1 (Reference)	12.5 (11.6-13.5) ***
Adjusted HR ^$^ (95% CI)			1 (Reference)	9.04 (8.22-9.94) ***

^$^ Multivariable analysis included age, monthly income, urbanization level, and comorbidities of schizophrenia, alcohol-related illness, anxiety, mental disorders, insomnia, diabetes mellitus, hypertension, hyperlipidemia, chronic obstructive pulmonary disease, coronary artery disease, stroke, and cirrhosis; *** *p* < 0.001.
